# What works in implementing shared medical appointments for patients with diabetes in primary care to enhance reach: a qualitative comparative analysis from the Invested in Diabetes study

**DOI:** 10.1186/s43058-024-00608-6

**Published:** 2024-07-24

**Authors:** Jodi Summers Holtrop, Dennis Gurfinkel, Andrea Nederveld, Julia Reedy, Claude Rubinson, Bethany Matthews Kwan

**Affiliations:** 1 Department of Family Medicine, University of Colorado Denver School of Medicine, 12631 E. 17th Avenue, Aurora, CO 80045 USA; 2 University of Colorado Denver School of Medicine, ACCORDS, 1890 N Revere Ct, Aurora, CO 80045 USA; 3 Department of Social Sciences, University of Houston-Downtown, 1 Main Street, Houston, TX 77009 USA; 4 Department of Emergency Medicine, University of Colorado Denver School of Medicine, 12631 E. 17th Avenue, Aurora, CO 80045 USA

**Keywords:** Diabetes, PRISM, Primary care, Health care delivery, Implementation champions, Qualitative comparative analysis

## Abstract

**Background:**

Diabetes is a serious public health problem affecting 37.3 million Americans. Diabetes shared medical appointments (SMAs) are an effective strategy for providing diabetes self-management support and education in primary care. However, practices delivering SMAs experience implementation challenges. This analysis examined conditions associated with successful practice implementation of diabetes SMAs in the context of participation in a pragmatic trial.

**Methods:**

Mixed methods study using qualitative and quantitative data collected from interviews, observations, surveys, and practice-reported data, guided by the practical, robust implementation and sustainability model (PRISM). Data were analyzed using qualitative comparative analysis (QCA). Successful implementation was defined as meeting patient recruitment targets (Reach) during the study period. Participants were clinicians and staff members from 22 primary care practices in Colorado and Missouri, USA.

**Results:**

The first necessary condition identified from the QCA was the presence of additional resources for patients with diabetes in the practice. Within practices that had these additional resources, we found that a sufficiency condition was the presence of an effective key person to make things happen with the SMAs. A second QCA was conducted to determine conditions underlying the presence of the effective key person (often performing functions of an implementation champion), which revealed factors including low or managed employee turnover, a strong baseline practice culture, and previous experience delivering SMAs.

**Conclusions:**

Identification of key factors necessary and sufficient for implementation of new care processes is important to enhance patient access to evidence-based interventions. This study suggests that practice features and resources have important implications for implementation of diabetes SMAs. There may be opportunities to support practices with SMA implementation by enabling the presence of skilled implementation champions.

**Trial registration:**

Registered at clinicaltrials.gov under trial ID NCT03590041, registered on July 18, 2018.

**Supplementary Information:**

The online version contains supplementary material available at 10.1186/s43058-024-00608-6.

Contribution to the literature

- Diabetes Shared Medical Appointments (SMAs) are an evidence-based approach recommended for providing diabetes self-management support and education in primary care. However, implementation challenges exist for complex, multi-component interventions such as SMAs.
- Qualitative Comparative Analysis allows for investigation of multiple data sources and paths to successfully attain an outcome. Using this method allows us to review implementation and outcome data from a large cluster-randomized pragmatic trial to explore single or multiple factors that in combination may lead to successful implementation.
- These results build upon previous literature supporting key factors for success for both SMAs and implementation science more broadly, including considerations for personnel and resource needs prior to planning interventions such as SMAs.

## Introduction

Diabetes is one of the most prevalent chronic diseases in the United States, with type 2 diabetes affecting more than 10% of the adult population [1, 2]. Associated with significant morbidity and mortality, type 2 diabetes has a high personal and economic cost to people living with the disease, their families, and society [3, 4]. Diabetes can be managed – and negative outcomes can be mitigated – through diabetes self-management support and education (DSME/S) [5–7]. Shared medical appointments (SMAs) are a recommended approach to providing DSME/S in primary care [8–10], with a systematic review and meta-analysis showing diabetes SMAs lead to clinically significant improvements in glycemic control [11].

Diabetes SMAs are complex, multi-component interventions requiring systematic change at the patient, provider, practice, and system level. Primary care practices deciding to adopt SMAs face challenges with implementation and sustainment [12]. Logistical challenges include availability of staff (e.g., skilled facilitators), billing and reimbursement, and patient participation barriers such as schedules, transportation, and cost [12]. SMAs often require new workflows, systems, and processes of care, which may require new and different resources, care team training to enhance knowledge and skills, reimbursement and funding models, and buy-in from practice leadership. Successful diabetes SMA implementation benefits from implementation champions [13], time, financial, and staff support from leadership [13, 14], fit between SMA features and patient and organizational characteristics [14], and motivation among patients and care team members [14]. However, successfully implemented diabetes SMAs in the literature tend to be heterogenous in that they have different implementation components (e.g., personnel involved, practice types, patient populations, etc.).

Thus, despite strong evidence that diabetes SMAs are an effective, patient-centered approach to providing DSME/S in primary care [10, 11, 15, 16], there is limited understanding of what factors are important for practices in implementation and sustainment of this complex behavioral intervention. What contextual factors are present in practices that are able to successfully implement diabetes SMAs? Are there characteristics of those practices or how they went about implementation that were important to improving reach and effectiveness for patients? To answer these questions, we studied contextual factors and key implementation determinants in a diverse set of community primary care practices implementing diabetes SMAs in the context of Invested in Diabetes, a pragmatic, hybrid type 2 effectiveness-implementation trial [17].

Recognizing the challenges and complexity of organizational change in primary care practices [18], we used Qualitative Comparative Analysis (QCA) to conduct a rigorous exploration of the key factors needed for implementation of SMAs in primary care. QCA is a research method that draws upon qualitative and quantitative data to examine factors (called conditions) that may singly or in combination be associated with an outcome. QCA is especially useful for studies with a smaller sample size as it is based on Boolean, rather than linear algebra, and is not constrained by degrees-of-freedom as with traditional statistical analysis. A key feature of QCA is the ability to examine if there are multiple ways to achieve an outcome (principle of equifinality). Thus, it is a useful analytic method to study complex causality and the effects of context. This paper reports the results of two iterative QCAs conducted using implementation process and outcomes data from the Invested in Diabetes study. These results build upon reports of barriers and contribute to a detailed understanding of successful implementation of SMAs in primary care practices.

## Methods

### Design and implementation science framework

This analysis reflects a mixed-methods study design including qualitative and quantitative data collected in the Invested in Diabetes study. The Practical, Robust Implementation and Sustainability Model (PRISM), which includes the outcomes of the Reach-Effectiveness-Adoption-Implementation-Maintenance (RE-AIM) model, was used to inform assessment of context and diabetes SMA implementation and sustainability factors [19, 20]. PRISM includes multiple contextual domains: organizational and patient characteristics, organizational and patient perspectives on the intervention, implementation and sustainability infrastructure, and external environment. Multiple methods (described below) were used to assess both PRISM domains and RE-AIM dimensions, which were then used in the QCA to initially identify categories of contextual factors that may be appropriate conditions (see step 4 below). As a hybrid type 2 effectiveness-implementation study, SMA implementation was a co-primary outcome along with effectiveness (reported elsewhere). As defined by RE-AIM, implementation outcomes (published elsewhere) included Reach (the number, percent, and characteristics of patients participating in diabetes SMAs) [21], Implementation (cost, fidelity, adaptations [22]), and Maintenance (practice sustainability plans). The primary implementation outcome used in the QCA was Reach.

### The Invested in Diabetes study

The Invested in Diabetes study was a pragmatic cluster-randomized comparative effectiveness trial that compared two models of diabetes SMAs in 22 primary care practices in Colorado and Missouri [17]. Eligible practices were those interested in implementing diabetes SMAs, had existing self-described integrated behavioral health professionals (BHPs), and had at least 150 adult patients with diabetes. Practice characteristics are shown in Table 1. Participating practices included two types: Federally Qualified Health Centers (FQHCs) and practices that primarily served patients with commercial insurance. Practices were randomly assigned to either a standardized or patient-driven approach for delivery of diabetes SMAs using the evidence-based Targeted Training in Illness Management (TTIM) group DSME/S curriculum, which was first developed for a population with concurrent type 2 diabetes and serious mental illness [23] and adapted for primary care populations [17, 24]. Most practices were asked to recruit and provide complete data (i.e., pre and post patient surveys, session participation records, and electronic health records data) at least 60 adult patients with type 2 diabetes over the course of 2 years (four practices were smaller or joined the study later and were asked to recruit and provide complete data for 30 patients); the implementation period was extended to 3 years due to COVID-19 related delays. The Colorado Multiple Institutional Review Board (COMIRB; protocol #17–2377) approved this protocol as exempt human subjects research. The study was registered on clinicaltrials.gov under trial ID NCT03590041 on July 18, 2018.

**Table 1:** Characteristics of primary care practices participating in the Invested in Diabetes study

Practice Characteristics	Statistics
Practice type N (%)	
FQHC	12 (55%)
Non-FQHC	10 (45%)
Location N (%)	
Urban	17 (77%)
Rural	3 (14%)
Suburban	2 (9%)
Number of clinicians with prescribing privileges Median (Range)	8 (2–65)
No. Staff eligible to be health educator Median (Range)	2 (1–6)
Estimated number of diabetes patients Median (Range)	549 (90–4,000)
Latino patients making up > 10% of practice N (%)	10 (67%)
Racial/ethnic minority patients making up > 20% of practice N (%)	13 (87%)
Insurance Median % (Range %)	3 (19%)
Private insurance, FFS or preferred provider organization	11% (3–70)
Private managed care	11% (5–35)
Medicare	19% (2–60)
Medicaid	40% (2–63)
Other public insurance	3% (0–5)
Self-pay or uninsured	10% (0–92)
Unknown	0% (0–10)
Other	4% (0–100)

Implementation of SMAs in participating practices was guided by the Enhanced Replicating Effective Programs (REP) framework [24, 25]. First, program materials were adapted and packaged for delivery in primary care and translated into Spanish in partnership with patient and practice representatives. All practices had an initial training with involved personnel that included instruction on the curriculum and assigned SMA delivery model and group facilitation techniques, as well as training in the study protocol and data collection procedures. They received both a TTIM instructor manual tailored to their assigned SMA model, PowerPoint presentations, and patient materials. Practices received implementation support from practice facilitators (4–5 initial sessions for workflow and process planning plus ongoing assistance with troubleshooting and unplanned adaptations) and participated in a quarterly learning community throughout the project implementation period. Further details on the protocol, implementation strategies, and early adaptations are published elsewhere [17, 24, 26].

### Selection and operationalization of implementation outcome for QCA: reach

The implementation outcome selected for the QCA was Reach, traditionally defined as the number and proportion of eligible individuals that participate in a program relative to a specified denominator [27]. For this study, the denominator was a practice’s target goal for patient enrollment in SMAs (defined as adult patients with type 2 diabetes attending at least one SMA session and completing patient-reported outcomes measures that are part of the intervention). Reach was selected as the implementation outcome for the QCA for several reasons. Reach is objectively quantifiable with a single indicator value (compared to fidelity or adaptations, which can require more complex or subjective measures). Reach reflects a critical practice-centered implementation metric, given sustainability of SMAs depends on practices’ ability to match patient demand with available resources. Furthermore, our operationalization of Reach accurately reflects practice efforts required to successfully deliver the SMAs: to get patients to attend and to complete key aspects of the intervention.

### Data collection and instruments

Data for the QCA came from multiple sources, including SMA tracking spreadsheets completed by practice staff, surveys administered to patients and practice members, interviews with patients and practice members, practice facilitator calls with practice contacts, and observations of the SMAs. Data used for the QCA was collected throughout the duration of the study and intended for multiple analyses, including the QCA (Table 2).

**Table 2:** Data collection time points

Time period	Baseline	Midpoint	Endpoint	Ongoing
Defined as	Within one month of SMA launch at practice	Around 9 months after SMA launch at practice	Within 2 months of final SMA cohort completion	Throughout the SMA implementation period. Collected as part of or during SMAs
Data collected	• Baseline practice survey with practice manager • Practice member interviews • Practice member survey: Practice Culture Assessment	• Practice member survey: Relational Coordination Survey • Practice interviews	• Practice member interviews	• Practice attendance • Patient reported outcome measures (including Diabetes Distress Scale) • SMA observations • Fidelity assessment • Practice coach notes

### Practice SMA tracking spreadsheet

Practice members implementing the SMAs were trained on how to complete the SMA tracking spreadsheet, which included patient SMA session attendance, patient demographics, and the patient survey scores (described below—primary and secondary outcomes for the main trial). Practices delivered updated SMA tracking spreadsheets to the study team on a quarterly basis.

### Practice and patient surveys

There were two types of surveys. First, there were practice member surveys which assessed the PRISM contextual factors of organizational and patient characteristics, and implementation and sustainability infrastructure. They included a baseline characteristics survey (completed by one person in each practice) and an assessment of practice culture (Practice Culture Assessment, PCA, completed by all practice members), which were completed pre-intervention (baseline) [24, 28]. Team-based care was assessed using the Relational Coordination Scale (RCS) by SMA care team members at baseline, at 9 months into the implementation period (midpoint), and end of the implementation period (endpoint) [29]. Surveys were completed either on paper or electronically using REDCap depending on the preferred method of administration. Study data were collected and managed using REDCap electronic data capture tools hosted at the University of Colorado [30]. Mean PCA and RCS scores were calculated for each practice, and used as potential “conditions” in the QCA.

Second, patients completed patient-reported outcome measure (PROM) surveys as part of the intervention. Patients completed the PRO measures at the first and last sessions and included several validated survey measures including the primary patient-centered outcome, diabetes distress, as measured by the 17-item Diabetes Distress Scale (DDS-17); a practice’s mean baseline DDS-17 scores were used in the QCA as an indicator of a practice’s patient characteristics [31, 32]. PRO measure surveys were collected by practice staff either on paper or electronically; the SMA instructors manual provided guidance on how to integrate baseline PRO measures into tailoring the SMA curriculum to patient needs and then using follow-up PRO measures to reflect upon and celebrate progress at the final SMA session. Completion of these PRO measures was noted in the SMA tracking spreadsheet and used to calculate Reach, as described above.

### Key informant interviews

Interviews with participating practice members were conducted by trained and experienced qualitative researchers at baseline, midpoint, and endpoint. Each individual interview was approximately 60 min, and participants were purposefully selected based on role with the project. Participants included medical providers, health educators, behavioral health providers, and SMA coordinators (*n* = 3–7 per practice). Semi-structured interview guides assessed all PRISM contextual categories including organizational and patient characteristics, organizational and patient perspectives on the intervention, implementation and sustainability infrastructure, and the external environment. Questions focused on practice infrastructure and resources, implementation successes and challenges, adaptations, staffing, and perceived support for SMAs by leadership and others. Interviews were done in person and on Zoom, audio recorded and professionally transcribed. Transcripts were loaded into Atlas.ti version 9 (ATLAS.ti Scientific Software Development GmbH) for support with analysis.

### SMA observations

Research staff observed SMA sessions to capture PRISM contextual factors and RE-AIM outcomes including primarily the intervention itself, specifically fidelity to both study protocol and curriculum (i.e., personnel used, time of sessions, covered content, etc.) as well as elements of the facilitation style and group interaction. The sampling goal was to purposefully observe a meaningful diversity of groups across practices sites and sessions without bias in selection and within study resources. Thus, the study protocol determined was to use a purposeful random sampling plan [33]; to observe one randomly selected session at each participating practice per quarter over six quarters of the study implementation period; the goal was to observe at least one of each of the six curriculum sessions at each practice over the course of the study. The research staff documented fidelity using a structured template that contained checklists to track delivery of core components of the curriculum as well as time spent and presence of specified personnel and formed a total fidelity score. Narrative field notes provided description of the assignment of selected rating. The structured template was developed based on previous guides used by the research team, but adapted to reflect specific measures needed to track core components of the overall intervention and differences between study conditions (i.e., inclusion of a peer mentor). All staff conducting observations were trained on the use of the template, including use on a sample pre-recorded SMA session.

### Practice facilitator-practice contact scheduled communication

Practice facilitators assigned to each practice conducted regular, ongoing contact with key practice members throughout the implementation period. These contacts were often by phone, but sometimes by email or in person. Templates were used to signal key topics for discussion and were also used to document progress with implementation including challenges and changes made to improve implementation. While an initial four pre-implementation facilitation meetings followed pre-planned agendas, additional facilitator contacts that produced written field notes were from ad-hoc meetings or emails [24]. The number of facilitation meetings (and thus field notes) varied per practice. After each contact/meeting, the facilitator wrote their notes in a narrative form in a running document in a shared online platform.

### Analysis

#### Team and overall process

A core qualitative team analyzed the data. This team included the qualitative lead (JH), a physician researcher (AN), and two research assistants (DG and JR). All had intimate knowledge of the study protocol and previous qualitative data collection and analysis experience, and all conducted and/or analyzed the interviews. DG collected or otherwise maintained all other data. A series of steps were completed for the data analysis. These are organized in the steps below.

#### Step 1: data analysis for individual data collection methods

For survey data, average baseline PCA, midpoint RCS, and baseline DDS-17 total scores were computed at the practice-level. For the qualitative data, we used a grounded hermeneutic editing approach [34]. Data were examined for key concepts and then a code book was created based on the emergent data. Iterative passes through interview transcripts by different researchers resulted in a complete code book and additional team work created a calibrated set of responses using the codes for the transcripts across researchers. After coding was complete, quotation reports were created to summarize key thematic areas and create narratives related to each theme. Practice observation data were analyzed for fidelity to the curriculum and the study protocol. This included seeing how many observed sessions had appropriate personnel and went through all topics of the curriculum. Observer ranking of facilitator skills were averaged by practice. Practice facilitator notes were reviewed by the core team and summarized to include reasons for facilitation meetings, issues encountered during implementation, adaptations, and sustainability planning.

#### Step 2: summary analysis across data collection methods

Next a summary template was developed to house practice-level summary information across all data sources which provided a practice-by-practice accounting of all the factors considered key to analyzing for implementation. The summary template contained these sections: data about the practice’s SMA cohorts, code group summary from baseline, midpoint, and endpoint interviews, summary of practice facilitator field notes, observation summary results and survey results. An assigned qualitative core team member reviewed all of the data sources for each practice and completed the template with one completed for each practice. An overall impressions narrative summary was added, which explicitly included analyst impressions on factors which emerged as possibly related to successful implementation. Then another core team member checked over the completed template using the same data sources. Any discrepancies were discussed and rectified which sometimes required revisiting the data and considering new categories on the template for summarization. Discussions were held to consider the meaning and relevance of key thematic elements that were emerging from the data. Categories of summary data were presented to the larger research team for discussion and feedback, then refined and finalized for consistency and implementation considerations. These efforts allowed the core team to revisit the data, confirm, modify or refute initial impressions, and identify additional areas for further analysis. Throughout the entire analysis process, an audit trail documented the discussion and decisions made. These completed summary templates provided the essential information for the further analysis.

#### Step 3: calculation of QCA main outcome

We calculated Reach, the primary implementation outcome for the QCA, at the practice level using the SMA tracking spreadsheet data. The number of patients who attended at least one session and had recorded PROM scores for both the first and last SMA sessions was divided by the target enrollment goal for each practice (30 or 60 patients, which varied by practice size and/or when they joined the study). Although the outcome is not a condition, it still needs to be calibrated for calculation of condition contributions to that outcome. The outcome was calibrated as a fuzzy set using the direct method (lower threshold = 0.329, crossover point = 0.669 and upper threshold = 1.0. We describe the calibration process in step 4.

#### Step 4: key conditions identified and condition calibration determined/table created

Given the choice of Reach as the implementation outcome of interest, we selected possible candidate factors (in QCA language, “conditions”). Using the summary templates and emergent key themes in Step 2, the team considered PRISM contextual factors and the domains they covered to identify candidate QCA conditions. Then within these domains, the team reviewed all the data to identify the most frequent and influential factors related to Reach. The explanatory conditions included those emergent from the qualitative analysis (such as leadership buy-in), practice characteristics (such as practice size), and other factors. The qualitative team then reviewed the conditions in multiple passes to highlight and group categories consisting of similar dimensions, which were then reviewed and approved by the PIs and practice facilitators. The process of corroborating/legitimating occurred through review of existing literature and seeking out associated experiences to confirm or refute insights from the analysis. After initial analysis identified data to support one theme or interpretation, particular effort was devoted to finding negative or disconfirming evidence. During our discussions, we recognized that some conditions described how the SMAs were implemented within a given practice while others described general characteristics of the practice itself.

Table 3 describes the explanatory conditions included in the analysis and how they were calibrated. In QCA, conditions may be scored as either “crisp” or “fuzzy,” depending upon the nature of the underlying measure. A crisp (dichotomous) condition has a score of 1 when a practice exhibits the characteristic and 0 when it does not. For example, a practice either is or is not an FQHC and therefore is represented as crisp. Fuzzy conditions are used when it is possible for practices to have partial set membership: be “more in than out” of a set (0.75) or “more out than in” (0.25) (ex: For the condition *Turnover*, if a practice had major turnover at leadership level but not in roles that were involved in implementation, shared medical appointments may be considered to be minimally to moderately affected and they may be more in the set of no turnover than out of the set (0.75)). We used the scoring step as an opportunity to refine condition definitions. For example, originally, we had the condition “key person”, which signified a set person who was tasked with implementing the intervention. This role was typically assigned at the launch of the intervention, and since having this point person was somewhat required for the study, we did not find enough variability in this condition (i.e., every practice would assign a key person), making it not useful for QCA. Upon reviewing the qualitative data, we realized that practices with an identified key person did not describe them as successful unless that person had dedicated protected time to implement the project, were knowledgeable around the organizational structure of the practice, and able to effectively problem solve implementation issues. We refined our definition to be a “effective key person”, which was defined as someone who had dedicated time and was capable of navigating practice SMA implementation challenges. We also allowed for degrees of inclusion in this condition if for example a key person had only some of these characteristics, or was significant in this role.

**Table 3:** Qualitative comparative analysis conditions and scoring

Condition	Definition	Data source	Scoring
**Conditions rated as “crisp” (0 vs. 1)**			
FQHC	Practice classified as an FQHC	Baseline practice survey	1 = FQHC; 0 = non-FQHC
Previous SMA experience	Any prior experience of group visits reported by practice	Baseline practice survey	1 = previous experience; 0 = no previous experience
Study condition	Practice in the patient-driven or standardized condition	Project details	1 = patient-driven condition; 0 = standardized condition
Practice facilitator	Which of the two practice coaches was assigned to the practice	Project details	1 = practice facilitator 1 (RW); 0 = practice facilitator 2 (JR)
Relational Coordination survey	Mean midpoint relational coordination score (poor vs OK vs good coordination)	Relational Coordination Survey	1 = good/OK coordination; 0 = poor coordination
Cost to patients	Efforts to reduce financial barriers for patients (beyond reducing provider visits)	Practice interviews, practice coach notes	1 = cost saving measures described; 0 = no measures described
Mean Diabetes Distress at baseline	Mean pre-SMA Diabetes Distress score of patients	Diabetes Distress Scale	1 = low distress; 0 = moderate/high distress
**Conditions rated as “fuzzy” (0, .25, .75, 1), usually involving qualitative assessment**^**a**^			
Turnover	Lack of extensive turnover of personnel that affected implementation staff	Practice interviews, practice coach notes	1 = No issues with turnover for project; 0 = extensive issues with turnover for project
Diabetes resources at baseline	Extra resources for diabetes care (beyond BHP) available before Invested in Diabetes (i.e., visits with nutritionist, diabetes educator, etc.)	Practice interviews, practice coach notes	1 = extensive diabetes care resources available; 0 = only standard care available
Facilitator skill	How good was facilitator during observations, average rating by observer	Observation data	1 = score of 4–5; 0 = score of 1–2. Score of 3–3.9 assessed qualitatively
Effective key personnel	Presence of effective key staff member, defined as one who has dedicated time for the implementation capable of navigating practice challenges	Practice interviews, practice coach notes	1 = effective key person involved; 0 = no person involved
Provider or leadership support	Indication that providers or clinic leadership support SMAs in a meaningful way (i.e., payment structures, approving admin support, etc.)	Practice interviews, practice coach notes	1 = clearly demonstrated support; 0 = no support
Continued prescribing provider involvement	Continued involvement of providers in SMAs, even if reduced by practice or conducted asynchronously. Need to have required at least 1 visit	Practice interviews, practice coach notes	1 = continued visits; 0 = discontinued visits
Practice culture at baseline	Practice Culture Assessment scores at baseline completed by entire practice	Practice Culture Assessment	1 = .75 + ; .75 = .65-.74; 0 = < .65
Fidelity to protocol	Fidelity to shared parts of Invested in Diabetes protocol	Fidelity assessment	80% + = 1; 70–79% = .75; < 70% = 0
**Conditions used as continuous**			
Number of patients with diabetes	Number of patients with diabetes in patient roster	Baseline practice survey	Count

^a^Unless otherwise explicitly defined, “fuzzy” conditions with a score between 0 and 1 indicate a practice that is close to, but not meeting the condition. A score of .75 indicates a practice that is closer to being included in the set of practices with the condition, while a score of .25 indicates a practice that is closer to not being in the set of practices that have the condition

#### Step 5: scores assigned and placed in the table for each practice

Once the conditions were identified for review and scoring, the analysis team utilized this system to score each practice for each condition with the results included in a table (or matrix), with the rows of the matrix enumerating the practices and the columns enumerating the explanatory conditions. Two researchers reviewed the practice summary from step 2 and independently assigned a score to each condition for each practice using Table 3 as a guide. Scores were shared and resolved through discussion including going back to the practice summaries and primary data, recalibrating if necessary, and determining each practice's final scores. Overall, scores were in high alignment. At this stage, practices who shared a centralized SMA delivery staffing model (*n* = 4) were merged into two practices (including summing their outcome goal) as we could not separate data for certain conditions; two practices that completed only a single SMA cohort were excluded from QCA analysis due to a high amount of missing data. This resulted in a completed table as depicted in Table 4 with 18 practice units.

**Table 4:** QCA condition inputs scored by practice

Condition	FQ01/08	FQ02/09	FQ03	FQ04	FQ05	FQ06	FQ10	FQ11	FQ12	PP01	PP04	PP06	PP07	PP08	PP09	PP10	PP11	PP13
**Crisp conditions**																		
FQHC	1	1	1	1	1	1	1	1	1	0	0	0	0	0	0	0	0	0
Previous SMA experience	1	1	1	0	1	0	1	1	1	0	1	1	1	1	1	1	1	0
Study condition	0	1	0	1	1	0	1	1	0	0	0	1	1	0	0	1	0	1
Practice facilitator	0	0	0	0	0	0	1	1	1	0	0	0	1	1	1	1	1	1
Relational Coordination survey	1	0	0	1	1	0	1	0	0	0	0	0	1	1	0	1	0	0
Cost to patients	0	0	1	1	1	0	0	0	1	0	0	0	0	0	0	0	0	0
Mean Diabetes Distress at baseline	0	0	0	0	0	0	1	0	1	1	0	0	1	0	0	1	0	0
**Fuzzy conditions**																		
**Turnover**	1	1	0	0	0.25	0.75	0	0	0.75	0.25	1	0	1	1	1	1	0	0
Diabetes care at baseline	1	0.75	1	0	0.75	0.75	1	1	1	0	1	1	1	1	1	1	1	1
SMA facilitator skill	1	1	1	1	1	0.25	0.25	0.25	1	1	0	0	0.25	1	0.25	0.25	1	0
Key personnel	1	1	1	1	0.75	1	0	0.75	1	0.75	1	0	1	1	1	1	0	0
Provider or leadership support	0	0	0.75	0	0.75	0.25	0	0	0.75	0	0.75	0.25	1	1	1	0.75	0	0
Continued prescribing provider involvement	1	0.75	1	0	1	0	0.75	1	1	1	1	1	0	0	0	0	0	0
Practice culture at baseline	0.75	1	0	0.75	0	0.75	1	0.75	0	0.75	1	0.75	0.75	1	0.75	0.75	0.75	1
Fidelity to protocol	0	0	1	0	0	0.75	0	0	0.75	0	0	0.75	0	0	0.75	0	0	0
**Continuous conditions**																		
Number of patients with diabetes	1222	5200	430	300	547	800	90	702	576	550	967	500	890	440	421	648	2112	484

#### Step 6: conducting the QCA

To complete the QCA, we used the standard protocol, as outlined by Rihoux and Ragin [35] and Mello [36] which included necessity and sufficiency analyses. Using the data from the matrix we created in step 5, the analyses were conducted using Kirq (https://grundrisse.org/qca/download), a publicly-available software package for QCA developed by co-author CR, and fs/QCA (https://fsqca.com), developed by Charles Ragin and Sean Davey.

### Definition of terms and interpretation of results

As QCA seeks to identify necessary and sufficient conditions related to the presence of an outcome, we first conducted a necessity analysis including all of the conditions. A unique benefit of QCA is that it is sensitive to issues of causal complexity (that multiple conditions may operate in tandem) and equifinality (that there may be multiple pathways or "recipes" to realizing an outcome). A necessary condition is a condition (or combination of conditions) that must be present for the outcome to occur; its absence prevents the outcome from occurring. Following necessity testing, we then completed tests of sufficiency. A sufficient condition is a condition (or combination of conditions) that, when present, ensures that the outcome will occur. Necessity and sufficiency can be imperfect: it is perfectly appropriate to identify a particular combination of conditions as "almost always" necessary or sufficient.

The degree to which a given condition or combination of conditions is necessary and/or sufficient for the outcome is measured by two goodness-of-fit metrics: consistency and coverage. Ranging between 0.0 and 1.0, consistency measures the strength of the necessary or sufficient condition. Established standards for QCA use thresholds of >  = 0.9 to establish necessity and >  = 0.8 to establish sufficiency. Coverage also ranges between 0.0 and 1.0 and measures empirical prominence; recipes with higher coverage scores explain a greater fraction of the outcome than recipes with lower coverage scores. A low coverage score may or may not indicate that the necessary or sufficient condition is trivial. There are not standard coverage scores for establishing necessity or sufficiency: a condition that explains only a small fraction of cases may still be substantively important.

## Results

### QCA #1: explaining practices who met the outcome (Reaching patient completion goals)

The results of the necessity analysis are presented in Table 5. Using the conventional consistency threshold of 0.9 [37], two conditions were identified as mathematically consistent with necessity: diabetes care at baseline (ncon 0.95, ncov 0.69) and the presence of an effective key person (ncon 0.95, ncov 0.80).

**Table 5:** Necessity Results for Explaining the Presence of Successful SMA Patient Recruitment and “Completion”

Term	Necessity Consistency (ncon)	Necessity Coverage (ncov)
Successful Key Person	0.95	0.80
Diabetes Care at Baseline	0.95	0.69
Solution	0.90	0.92

Necessity consistency reports the degree to which those practices possessing the respective condition also exhibit the outcome; necessity coverage reports the degree to which practices possessing the necessary condition also possess the outcome. Solution consistency and coverage reports consistency and coverage for practices that possess either or both of the necessary conditions. Only those conditions with a necessity consistency score greater than 0.9 are presented

It cannot be concluded that necessity exists based merely upon a strong consistency score; a corresponding theoretical justification must also be established. For our analysis, having additional diabetes care resources as a necessary condition made sense: Practices who invest in diabetes care (condition defined in Table 3) will likely have the appropriate infrastructure (i.e., personnel for recruitment, provider/educator staff that provide services beyond 1:1 visits, etc.) while those who do not invest in diabetes care likely would not. A prepared and adequately resourced practice is more likely to be successful at implementing a new diabetes intervention, particularly one as complex as a diabetes SMA.

The “effective key person” factor possessed a necessity coverage score of 0.80, in addition to its high consistency score. QCA's measure of necessity coverage is the same as its measure of sufficiency consistency, for which the established threshold is 0.80. The results of the necessity analysis therefore revealed that the forthcoming sufficiency analysis would identify the presence of a key person as mathematically sufficient on its own for the outcome (i.e., no other conditions were needed to explain the outcome). When both consistency and coverage scores are high, conditions may be interpreted as consistent with necessity, sufficiency, or both. This is also true for the overall solution, which exhibited high consistency and coverage (0.90 and 0.92, respectively). One interpretation of these results could be that the presence of these conditions was individually-necessary and jointly-sufficient for the outcome's occurrence. This made sense to us: implementation researchers have repeatedly found that institutional resources–including the presence of a person who serves as an implementation champion and not only advocates for and implements the intervention, but is also well connected and able to navigate the practice setting effectively–is critical to the success of novel healthcare initiatives [38]. Our results aligned with such findings, indicating that successful SMA patient completion will be realized when a practice with pre-existing diabetes care investment is able to assign or recruit a successful key person to oversee and facilitate the program.

### QCA #2: explaining the presence of an effective key person

Confronted with these results, the question then became, "What explains the presence of an effective key person?" It was clear we were investigating a causal chain, and thus began another round of QCA. Necessity testing did not identify any conditions as required for having an effective key person and we turned to the sufficiency analysis.

**Fig. 1:**
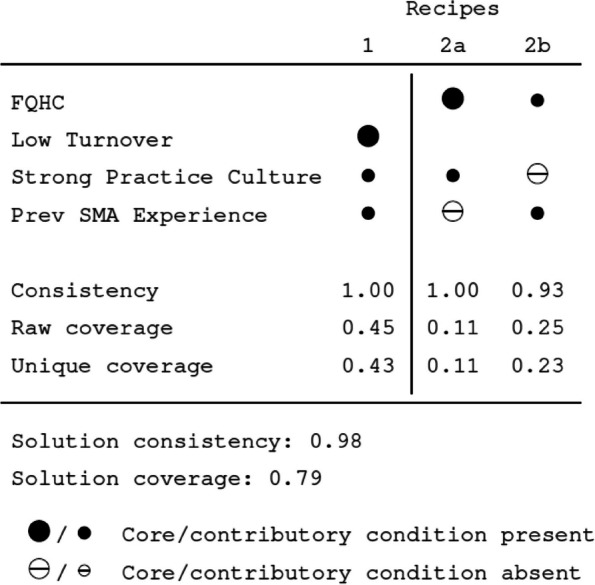
Fiss chart describing three combinations for presence of a key person condition

Using the same explanatory conditions, our sufficiency analysis identified four conditions that combined to form three different causal pathways ("recipes") describing practices that were able to recruit or retain an effective key person to manage and implement the SMA intervention. The Fiss configuration chart (Fig. 1) presents these results and associated consistency and coverage scores. One recipe describes practices with low staff turnover. Such practices also needed to possess a strong practice culture and prior experience with group visits. This describes a well-run practice that has invested, competent staff with prior expertise in knowing what they need to implement a diabetes SMA within their practice.

**Fig. 2:**
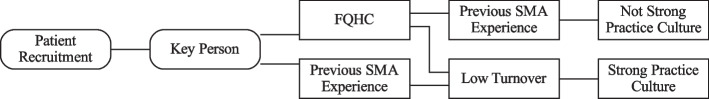
Branching diagram of sufficiency combinations for presence of key person condition

Two other recipes describe presence of an effective key person in FQHCs. For the participating FQHCs, the presence of an effective key person depended upon having either a strong practice culture (but not prior SMA experience) or prior SMA experience (but not a strong practice culture). Prior research has shown that FQHCs may be more acquainted with running innovative and more labor-intensive behavioral interventions, and be incentivized to do so via federal financial support, than commercial pay practices, who may be more adept in providing patients with 1:1 clinician visits [39]. As such, they may be better prepared to implement complex interventions such as diabetes SMAs. Figure 2 shows the causal pathway identified to successful SMA implementation.

## Discussion

In this study, we found that having increased baseline diabetes resources (e.g., resources beyond physician and BHP availability, such as availability of 1:1 nutritionist or health coach sessions) formed the first level of understanding regarding practice success in implementing SMAs. This is reasonable because practices likely need to have a certain amount of resources and support for diabetes care for the implementation of SMAs to occur effectively. In addition, practices who at baseline were devoting ample resources to patients for managing diabetes care could be viewed as ones who were motivated to help patients successfully change behaviors and who were in positions where they could add these additional services without overwhelming the business side of the practice. This is consistent with other research on readiness and capacity to make changes [40].

Once diabetes resources were in place, our next main sufficiency condition was having an effective key person who could “make it happen.” A dedicated, knowledgeable, and motivated key person who had sufficient protected time to implement the SMAs was consistently referenced in our qualitative findings as someone who was indispensable to making sure the project ran smoothly. In fact, the definition of our effective key person was similar to that of an “implementation champion”, someone who is motivated and supported to champion a project or cause within a project. Our results demonstrate the importance of defining personnel roles and responsibilities and the value of an implementation champion [38, 41] – including for diabetes group visits [42]. Understanding what enabled a key person to be successful and capture the needed characteristics revealed three main “recipes” for the practice-level conditions that set practices up for success: various combinations of low staff turnover, strong practice culture, and/or previous experience running SMAs. This first grouping of conditions indicates that the practices who were most able to employ an effective key person did not have to worry about their key person or support personnel leaving the practice, which decreases strain to all staff (yielding lower turnover), such that personnel felt positively about working at the practice and felt supported taking on additional roles (strong practice culture), and had experience with quickly identifying and overcoming any barriers to SMA implementation that might be required (previous SMA experience). That is, these practices were primed for success. Our other two recipes, however, described FQHCs with fewer supporting conditions and here an effective key person nevertheless emerged when practices had only strong practice culture and low or controlled turnover (but not previous experience with SMAs) or previous experience with SMAs (but not strong practice culture). These practices succeeded despite possessing fewer advantages than those described by the first recipe. We posit that FQHC practices (in general but in our sample specifically) may be more accustomed to offering patient resources for diabetes care outside of traditional 1:1 clinician visits, as they have federal financial incentives that support patient health outside of provider-based fee-for-service models [39]. It may be the case then that FQHCs may be better set up to succeed in delivering SMAs even in non-optimal circumstances.

Our findings, in conjunction with known literature on practice resources and implementation champions, point to the need for practices who wish to implement complex behavioral interventions, to invest first in the organizations’ personnel. Comparative-effectiveness of the two SMA delivery models revealed little if any difference between standardized and a more expansive, “patient-centered” approach. Further findings from the QCAs show that the factors that seemed to join together to create the sets of successful practices described practices that were willing and/or able to invest in resources for patients (diabetes resources at baseline) and ensure a diabetes SMA champion could emerge (successful key person). We acknowledge that this may be difficult to achieve, particularly in practices that rely on fee-for-service reimbursement for their primary payment mechanism. We posit that this is one of the reasons that we found that our FQHCs, who have other financial streams, had more ways of reaching our successful practice recipe than commercial pay practices. The policy implications from these findings reflect the need for practices to have the resources (reimbursement and/or funding and well as personnel with training available) for both implementation of SMAs as well as having adequate diabetes care resources overall.

Our findings mirror other studies showing the importance of champions in behavioral health interventions. These studies emphasize that “champions are important” for practice change [38] and describe characteristics of effective champions [38, 42],, however, despite being one of the most commonly employed and successful implementation strategies in clinical settings [43, 44], there remains a lot to be learned about the best ways to support and prepare clinical champions [44]. There are few champion training programs; those that exist primarily focus on educational workshops and online training related to the evidence-based intervention and implementation and leadership strategies [42].

A major opportunity presented in this project was gaining an understanding of the circumstances under which practices were able to implement and sustain SMAs. Reach is important for practices to justify the use of limited personnel resources (i.e., staffing allocations, clinician time) and to bill at adequate levels of reimbursement. Attracting patients to participate in SMAs was a recurrent issue expressed by participating practices. Accordingly, the Reach domain from RE-AIM was the main implementation outcome selected for the QCA, which provided insights on “recipes” or combinations of important conditions for practice contextual factors associated with meeting patient recruitment targets. Reach is typically evaluated using the percentage of patients who participate in an intervention among a useful denominator – as our practices were contracted to have a set number of SMA participants (30 or 60), and there was high variability in attaining the outcome [20], this became a straight-forward outcome to measure for the QCA.

There are study limitations. Qualitative work inherently is not meant to be inferential; it is intended to provide insight into what the selected group of participants might be experiencing or perceiving. Thus, this population of practices from selected Colorado and Missouri practices may not be applicable to other populations. Even though we had very few practices decline participation, it is possible that some selection bias may have created a sample that was predisposed toward successful implementation of SMAs. We removed two practices who ended up not being successful from our dataset because of data missingness, which may have further contributed to this bias. Our functional definition of reach as the QCA outcome given that we were not able to examine those patients offered versus those enrolled and instead measure program completers as reach may also have affected our results such that we might have identified other factors if we had a true measure of reach. The researchers completing this work utilized multiple methods to validate the results including triangulation across researchers and multiple analysis methods, however, the results may have reflected bias or misinterpretation of the stories shared in the interviews or gathered from other data collection methods.

## Conclusions

For evidence-based DSME/S to reach patients in the form of SMAs, implementation at the primary care practice level needs to occur. Understanding what factors are important in successful implementation may provide a useful guide map for primary care practices as they begin implementation in their own settings. This research demonstrates the importance of a baseline level of personnel and other resources devoted to diabetes care and an activated and supported person to facilitate delivery of SMAs. Although practice culture and employee turnover are challenging factors for many practices, they do contribute to the ability of the practice to take on new initiatives. Therefore, careful consideration of the practice environment may be wise before endeavoring to begin implementing diabetes SMAs and likely any new intervention that requires considerable resources and processes.

### Supplementary Information

Supplementary Material 1.

## Data Availability

The data generated and analyzed during this study are in the form of qualitative interview transcripts and field notes. These data are potentially available from the corresponding author under specific conditions upon reasonable request. The data are de-identified and stored in a password secured database at the University of Colorado.

## Abbreviations

DSME/S: Diabetes Self-Management Support and Education
SMAs: Shared Medical Appointments
QCA: Qualitative Comparative Analysis
PRISM: Practical, Robust Implementation and Sustainability Model
RE-AIM: Reach-Effectiveness-Adoption-Implementation-Maintenance Model
BHPs: Behavioral Health Professionals
FQHCs: Federally Qualified Health Centers
TTIM: Targeted Training in Illness Management
REP: Replicating Effective Programs Framework
PCA: Practice Culture Assessment
RCS: Relational Coordination Scale
PRO: Patient-Reported Outcome
DDS-17: Diabetes Distress Scale
PIs: Principal Investigators
