# Supplementary material for: What works in implementing shared medical appointments for patients with diabetes in primary care to enhance reach: a qualitative comparative analysis from the Invested in Diabetes study
Source: Implement Sci Commun. 2024 Jul 24;5:82. doi: 10.1186/s43058-024-00608-6 (PMC11267890; doi:10.1186/s43058-024-00608-6)
Supplement: Supplementary file 1 — Supplementary Material 1. [file 43058_2024_608_MOESM1_ESM.docx]

| FQHC | Turnover | Previous SMA experience | Practice Culture | N | Consist | Outcome |
| --- | --- | --- | --- | --- | --- | --- |
| True | True | True | True | 2 | 1.00 | True |
| True | True | True | False | 1 | 1.00 | True |
| True | True | False | True | 1 | 1.00 | True |
| True | False | True | True | 2 | 0.43 | False |
| True | False | True | False | 2 | 1.00 | True |
| True | False | False | True | 1 | 1.00 | True |
| False | True | True | True | 5 | 1.00 | True |
| False | False | True | True | 2 | 0.00 | False |
| False | False | False | True | 2 | 0.43 | False |

Supplemental Table 1: Truth table explaining presence of a key person‍
